# Sustainable Water Management in the Southwestern United States: Reality or Rhetoric?

**DOI:** 10.1371/journal.pone.0011687

**Published:** 2010-07-21

**Authors:** Robert M. Marshall, Marcos D. Robles, Daniel R. Majka, Jeanmarie A. Haney

**Affiliations:** The Nature Conservancy Center for Science and Public Policy, Tucson, Arizona, United States of America; Smithsonian's National Zoological Park, United States of America

## Abstract

**Background:**

While freshwater sustainability is generally defined as the provisioning of water for both people and the environment, in practice it is largely focused only on supplying water to furnish human population growth. Symptomatic of this is the state of Arizona, where rapid growth outside of the metropolitan Phoenix-Tucson corridor relies on the same groundwater that supplies year-round flow in rivers. Using Arizona as a case study, we present the first study in the southwestern United States that evaluates the potential impact of future population growth and water demand on streamflow depletion across multiple watersheds.

**Methodology/Principal Findings:**

We modeled population growth and water demand through 2050 and used four scenarios to explore the potential effects of alternative growth and water management strategies on river flows. Under the base population projection, we found that rivers in seven of the 18 study watersheds could be dewatered due to municipal demand. Implementing alternative growth and water management strategies, however, could prevent four of these rivers from being dewatered.

**Conclusions/Significance:**

The window of opportunity to implement water management strategies is narrowing. Because impacts from groundwater extraction are cumulative and cannot be immediately reversed, proactive water management strategies should be implemented where groundwater will be used to support new municipal demand. Our approach provides a low-cost method to identify where alternative water and growth management strategies may have the most impact, and demonstrates that such strategies can maintain a continued water supply for both people and the environment.

## Introduction

Although the concept of sustainability is widely touted as an ideal for urban growth policies, achieving sustainability can be difficult. Nowhere is this more evident than in the case of water and water uses. Sustainability in the realm of freshwater is generally defined as the provisioning of water for both people and the environment for generations to come [1]–[4]. Unfortunately, plans for urban growth rarely embrace this definition for water policy and instead focus almost entirely on the pursuit of new supplies to accommodate future growth without consideration of environmental water needs [5].

Conflict over water has become a hallmark of the southwestern United States, where large-scale water infrastructure projects in the 20^th^ Century facilitated rapid urban and economic expansion at the expense of the environment. As dams, diversions, and increased groundwater pumping modified hydrologic regimes throughout the western United States, surface flow diminished or disappeared altogether at some locations, and associated riparian and aquatic systems declined [6]–[8].

Populations and cities continue to expand in the western United States, which calls into question what might be done to promote sustainable water use moving forward. A crucible for these pressures is Arizona, where population is projected to double by 2050 [9] and streamflow depletion has been documented throughout the state [10], [11]. Seventeen of the state's 33 native fish species now have status under the U.S. Endangered Species Act [12]. Three fish species within the Colorado River Basin (Pahranagat spinedace, Las Vegas dace, Monkey Spring pupfish) have already been driven to extinction from human modification of aquatic habitats and introduction of non-native species [13]–[16]. Our analysis suggests there will be additional streamflow depletion and further species imperilment without actions to reverse current trends.

Using Arizona as a case study, we develop a scenario-based assessment approach as a tool for exploring how water management strategies could sustain water for both people and the environment. Our study area is experiencing some of the highest growth rates in the United States, and this growth is relying on the same groundwater that supplies year-round flow in our rivers. We present the first study in the southwestern United States that evaluates the potential impact of population growth and water demand on streamflow depletion across multiple watersheds.

## Materials and Methods

### Estimating River Base Flow

**Figure 1:**
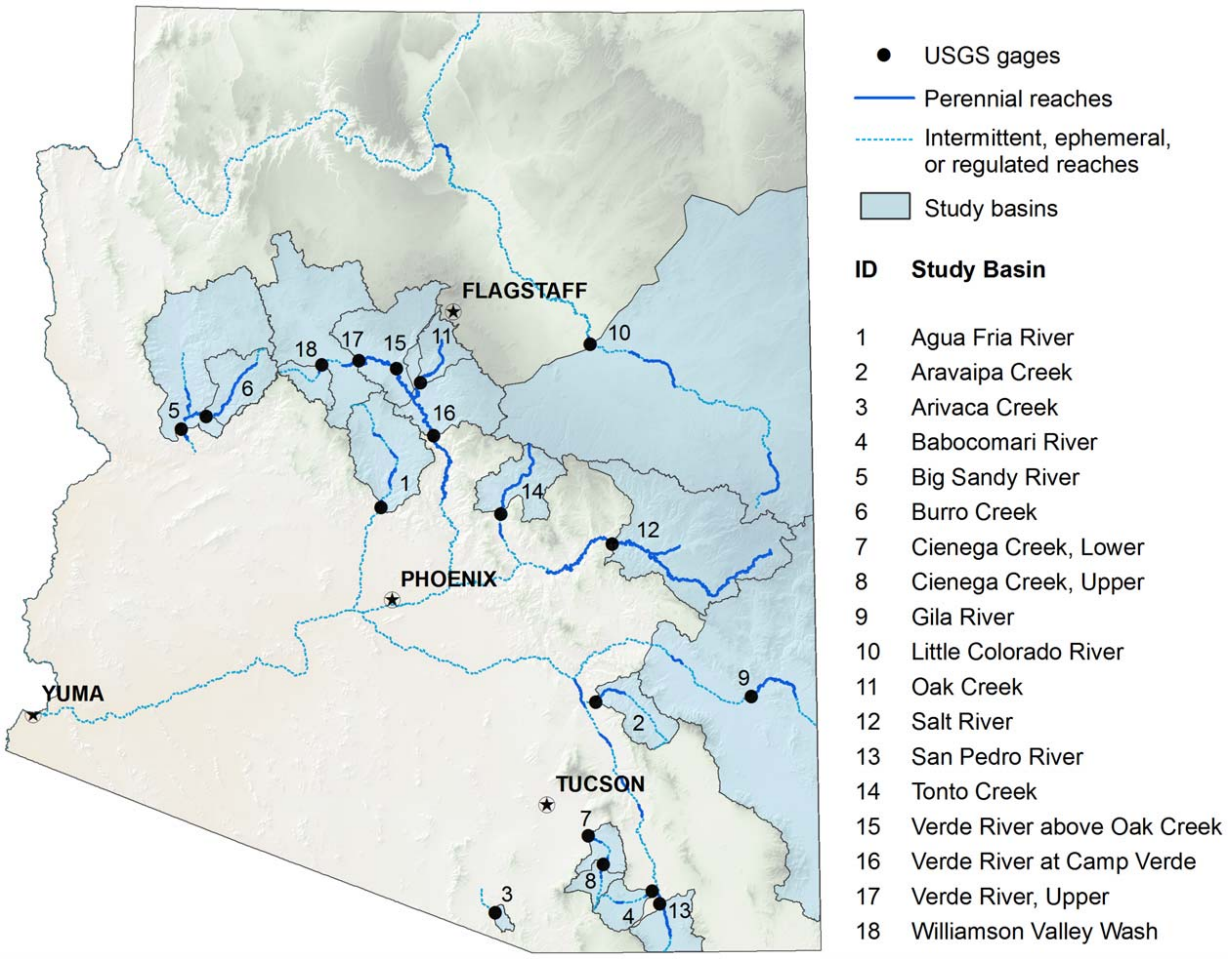
Locations of study watersheds and USGS gages.

Base flow is the proportion of surface flow that comes from groundwater discharge and supports year-round streamflow. To estimate base flow, we first inventoried locations where groundwater discharge still supports perennial streamflow across the state. We intersected the USGS streamflow gage data layer (http://waterdata.usgs.gov/az/nwis/rt) with a GIS layer of perennial streamflow [17] and selected 18 gages on unregulated perennial streams (Figure 1). We focused on gages with at least a 20-year data record, although for four gages we accepted a record of 7 to 10 years. We digitized the watershed area reporting to each selected streamflow gage using groundwater basin boundaries delineated by the Arizona Dept of Water Resources [18] as a starting point (Text S1). We researched published base flow values and evaluated several methods of base flow separation. We found that accurately separating base flow is difficult in areas of low total runoff [19]. We examined flow duration curves and daily mean flow hydrographs, and, where available, we compared published base flow values to median flow. From our analysis, we concluded that median streamflow approximates base flow for our study streams (Text S1).

### Estimating Current and Future Municipal Water Demand

We estimated municipal (i.e. all residential) water demand in the study watersheds by combining water use data from the Arizona Water Atlas [18], with current and projected population data obtained from the 1990 and 2000 Census [20] and Arizona Department of Commerce [9]. We calculated per capita water use rates (gallons per capita per day, GPCD) using watershed demand and population data over a recent 10 year period, 1996–2005. These rates were calculated for Arizona Department of Water Resources groundwater basins, within which the study watersheds are nested [18]. To calculate municipal demand in the year 2000, we multiplied GPCD rates by watershed populations derived from 2000 Census Block data (Table S1).

Because county-level population projections are spatially incongruent with our study watershed boundaries, it is not possible to calculate future demand without first estimating future population projections at a finer spatial resolution. To estimate future water demand, we multiplied year 2000 GPCD rates by population projections created using the raster-based Spatially Explicit Regional Growth Model (SERGoM) [21].

SERGoM allocates county-level population projections [9] for future decades (2010, 2020, 2030, 2040, 2050) to 100 m pixels within the study watersheds. Projected growth is not allocated to pixels classified as water, private protected lands, federal lands, or steep slopes >25%. SERGoM estimates future growth based on 3 basic steps. First, the model calculates past growth trends between the previous and current time step (e.g. 1990 and 2000) within 24 development classes. These classes are derived by combining 4 housing density classes (urban, suburban, exurban, rural) with 6 automobile travel-time classes (0–5, 5–10, 10–20, 20–30, 30–45, >45 minutes to the nearest urban or suburban core area). Second, the model distributes county-wide population projections to each 100 m pixel according to the relative growth rate of the pixel's development class. Third, the model recalculates development classes at each decadal time step, to allow for development classes to evolve or change as urbanization occurs (e.g. development of a new urban core).

### Water Demand Scenarios

We developed four population growth and water management scenarios. Estimates for Arizona's 2050 population have ranged from 8 million [22] to 16 million [23], with an estimate of 12.8 million by the latest Arizona Department of Commerce projections [9]. To accommodate this range of uncertainty and explore the effects of alternative water management strategies, we modeled municipal water demand in each watershed under four scenarios: 1) base – uses population estimates from Department of Commerce with constant GPCD; 2) high growth – increases population by 25% above base projection, 3) conservation – retains base population projection but reduces water demand 30% by 2050; 4) conservation and low growth – reduces water demand by 30% by 2050 and reduces population 25% below base projection. We selected 30% as a water conservation target because cities such as Albuquerque, NM and Long Beach, CA have successfully reduced their municipal water demand by at least that much [24].

### Comparing Base Flow to Water Demand

We compared base flow to municipal water demand in each of the 18 study watersheds to determine degree of streamflow depletion under equilibrium conditions when steady state conditions are reached for each projected water demand [25].

To assess the relative impact of municipal water demand on river base flow we used a simple index, the base flow demand index (BDI) where BDI  =  demand/base flow * 100. Although empirically-derived thresholds that predict species' persistence exist for some riparian plant species [26], most aquatic and riparian-dependent species in our study area lack such thresholds. In lieu of these thresholds, we present risk to base flow in relative terms by categorizing BDI values into 3 ranges: 
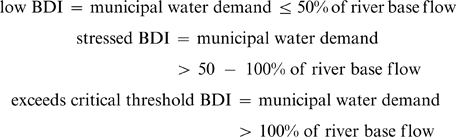



As an example of the practical implication of BDI values, a BDI of 100%, over time, would result in the complete de-watering of a river. Year 2000 baseline BDI values for the 18 watersheds examined as well as projected BDI for the 4 management scenarios are available as supplementary information (Table S1).

### Species Analysis

We identified 59 rare, plant and animal species (vertebrates and invertebrates) that occur within the study area and can be classified as obligate- or facultative- aquatic, wetland or riparian species (Table S2). First, we selected all rare species that have an extant population within the study watershed boundaries observed by an authoritative source since 1975 [27]. These are species or subspecies that are either globally rare (NatureServe Global Conservation Rank of G1-G3 for species, T1-T3 for subspecies) or are listed under the US Endangered Species Act (endangered, threatened, candidate, proposed, special concern, similarity of appearance). Second, we identified species that are obligate- or facultative- aquatic, wetland or riparian species using standard references [28], [29] and expert review. From this list of species, we used a regional conservation database to compare the number of aquatic, wetland or riparian species within our study area that have status under the U.S. Endangered Species Act to the number found within the Colorado River Basin [30].

## Results

**Figure 2:**
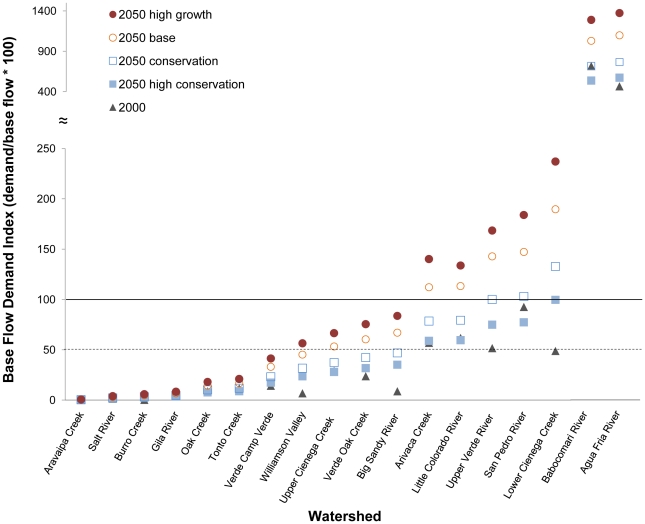
Base flow demand index (BDI) for 18 watersheds for the year 2000 and across four future scenarios. BDI is calculated as the percentage of municipal water demand to river base flow within a watershed. The solid horizontal line indicates the threshold where demand exceeds 100% of base flow; the dashed line delineates the transition from low BDI (≤50% of base flow) to stressed BDI (>50%–100% of base flow). See text for description of scenarios.

Our models indicate that in 2000, municipal water demand had already exceeded 100% of base flow in two watersheds, and was nearly 100% of base flow in one other (Figure 2). On the other end of the demand spectrum, there are seven watersheds where not only is current BDI low,demand is projected to remain in the low BDI range under any growth/water management scenario (Figure 2). The critical opportunities for management are found in the intermediate watersheds, where growth/water management scenarios determine whether or not watersheds fall into the dewatered status or remain a sustainable resource for biodiversity and people. In particular, implementing either of the two conservation scenarios would prevent four watersheds from transitioning from the low to stressed BDI range (Williamson Valley, Upper Cienega Creek, Verde Oak Creek, Big Sandy River; Figure 2). Three of these watersheds have relatively low annual discharge, so communities in these areas have little water to begin with. One watershed ranks within the top five in annual discharge; it has a relatively large volume of water, but also a population projected to more than double by 2050 (Table S1).

In year 2000, demand in each of the five remaining watersheds was already approaching or within the stressed BDI range (Figure 2). However, implementing one of the conservation scenarios would prevent four of these systems from transitioning to the critical threshold BDI range (Arivaca Creek, Little Colorado River, Upper Verde River, San Pedro River; Figure 2).

**Figure 3:**
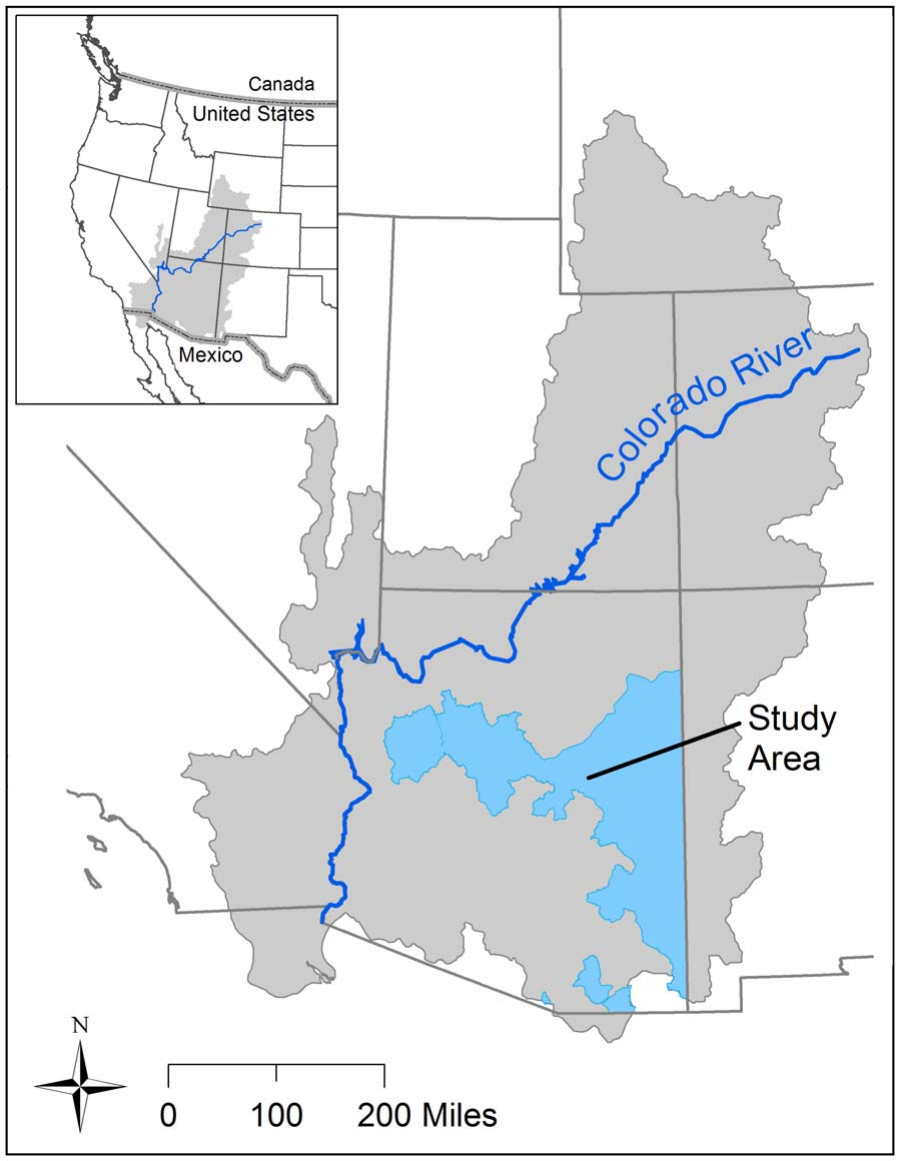
Regional significance of the study watersheds to species. Nearly one-half (45%) of the aquatic, wetland and riparian species that are listed under the U.S. Endangered Species Act in the Colorado River Basin occur in the watershed basins of this study. Inset: Colorado River Basin within North America.

The outcome of our modeled water demand/river flow relationships has important implications for plant and animal species that are dependent upon freshwater environments. While our study watersheds comprise only 11% of the area within the Colorado River basin (Figure 3), they host 45% of the aquatic, wetland and riparian species in the basin currently listed under the U.S. Endangered Species Act [30]. If current urban growth and water use trends follow our projections, more species will trend toward threatened or endangered status and, perhaps, extinction (Table S2).

## Discussion

In Arizona, natural perennial streamflow has already declined or disappeared completely at a number of locations due to human groundwater use [7], [10], [11]. Our study demonstrates that if actions are not taken to reserve a portion of river base flows for the environment, then at least seven other river systems will be de-watered over time and an additional four will experience substantial degradation. Although coarse, our scenarios illustrate how future risks can be reduced by implementing alternative growth and water management strategies. The question is how to do so in practice.

**Figure 4:**
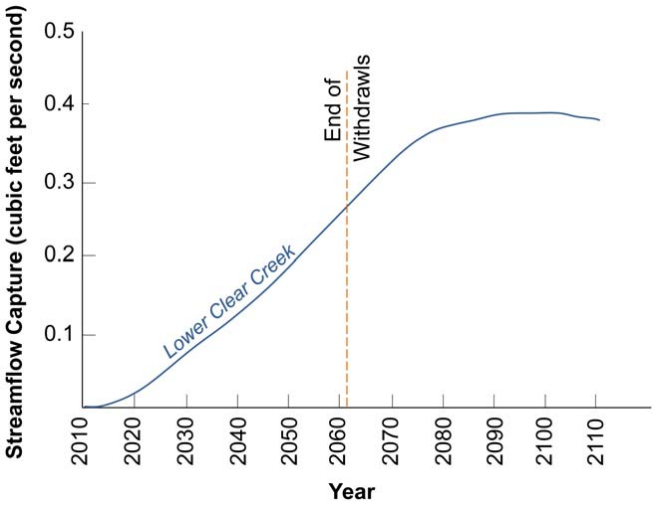
Cumulative effects of groundwater pumping on streamflow depletion even after pumping is stopped. In this case, from Lower Clear Creek in Arizona, streamflow continues to decline for nearly 30 years after pumping is stopped in 2060. Adapted from Alley and Leake (2007).

Renewable water supplies are limited in the Southwest. Even though Arizona's alluvial basin aquifers contain substantial amounts of water, it takes only a small fraction of use for effects to show up as river depletion [31]. Time lags associated with groundwater systems can extend the time frame of full depletion out decades (Figure 4) [32].

One challenge for any approach to sustainable water use in Arizona is that climate change will intensify existing difficulties of maintaining water supplies in an arid climate prone to drought. With climate change, surface flows in the southwestern U.S. will likely decline as temperatures continue to rise and evaporation rates increase. Colorado River flows are predicted to decline by 10–30% [33], and there is an 85% chance that flows in the Salt and Verde River basins will be reduced by 2050 [34]. Additionally, climate change may lead directly to reductions in base flows by reducing groundwater recharge [35], [36].

In addition to climate issues, there are regulatory and policy complications. The complexity of Arizona's laws and regulations guiding surface and groundwater use arose out of an attempt to bring equity to all water users. Water for the environment, however, was not considered in decisions regarding equity. In Arizona, surface water and groundwater are managed under different regulatory schemes and there is no legal recognition of the physical connection between the two. In the majority of our study area, groundwater use is unregulated and rivers can be de-watered through groundwater extraction.

Our current environmental policies and legal framework do not consider environmental water needs and, thus, are inadequate to protect flowing rivers. Planning is currently underway for water infrastructure projects to be implemented 20 or more years in the future. By the time those projects receive funding and are subjected to environmental compliance, further degradation and loss of river flows will already have occurred. With implementation of one or more conservation strategies now, impacts to streamflow can be reduced. As Figure 4 illustrates, if groundwater pumping continues, impacts to the river accumulate and cannot be immediately reversed if pumping is stopped altogether. Thus, mitigating project effects to endangered species 20 or more years in the future is not the same as acting now while we still have options in many watersheds to allocate water for the environment.

Fortunately, the discussion over water sustainability is already being reframed by communities that recognize the interdependence of the environment and economy. Many communities and regions around the world are implementing sustainable water management policies that address the needs of competing sectors while sustaining water for the environment. For example, Australia's constitution now mandates environmental flows as the first allocation [37]. Kansas [38], Michigan [39], and Massachusetts [40], among other states, have developed tools and regulatory mechanisms to limit new water uses such that existing users, including the environment, can maintain current condition. The Upper San Pedro River Partnership in Arizona has employed hydrologic and ecological models to support zoning overlays that encourage groundwater pumping more distant from the river [41] and recharge of treated municipal effluent near the river [42].

The underlying drivers in many of these examples are economic as much as environmental; in the era of sustainable development, would private investment flow to communities perceived as unsustainable? As our study shows, developing modest growth and water management strategies can ensure a continued water supply for people and rivers, and transform the rhetoric of sustainable water management into reality. The quicker we act, the more options we will preserve.

## Supporting Information

Text S1 Estimating river base flows. Contains detailed methods on how we estimated river base flow in the 18 watersheds studied.
(0.09 MB DOC)

Table S1 Population, base flow, and municipal water demand under four scenarios for eighteen river basins in Arizona. Provides baseline data used for estimating population growth, water demand, river flows, and the values used in scenarios.
(0.06 MB DOC)

Table S2 Vulnerable aquatic, riparian and wetland species found in 18 study watersheds in Arizona. Provides taxonomic and conservation data for the imperiled species found within our study watersheds.
(0.09 MB DOC)
