# Supplementary material for: Sustainable Water Management in the Southwestern United States: Reality or Rhetoric?
Source: PLoS One. 2010 Jul 21;5(7):e11687. doi: 10.1371/journal.pone.0011687 (PMC2908145; doi:10.1371/journal.pone.0011687)
Supplement: Text S1 — Estimating river base flows. Contains detailed methods on how we estimated river base flow in the 18 watersheds studied. (0.09 MB DOC) [file pone.0011687.s001.doc]

**Estimating River Base Flow**

Base flow is that part of streamflow originating from groundwater discharge; thus, base flow sustains year-round flow. Base flow is augmented by rainfall snowmelt and runoff during and following precipitation events. Base flow separation is a technique used to estimate the percentage of total annual streamflow originating from groundwater discharge. Published base flow values are few for streams in Arizona. An exception is the upper and middle Verde River watershed, where HYSEP base flow separation was conducted on USGS streamflow gage data [1]. We compared these published base flow values to median flow for those gages and found close correspondence (Figure S1).

Base flow separation techniques utilized in more humid regions are not very accurate in areas of low total runoff [2]. In arid regions such as the southwestern U.S., streamflow is typically small and reflects discharge from groundwater during the majority of the year. Flow duration plots for streams in the study area show a fairly flat line across exceedance values ranging from 10 to 90 percent, illustrating that these streams spend the majority of time in a base flow condition (Figure S2). This is especially true for the study streams with low total runoff (Figure S2A); these streams drain smaller and lower elevation watersheds, typically in southern and western Arizona. For the larger study streams, which drain larger and higher elevation areas in east-central Arizona and include a larger runoff component, flow duration plots show additional slope to the line, especially at the lower exceedance values, but are still relatively flat (Figure S2B). Thus, our flow duration plots indicate that median flow is a reasonable approximation of base flow for the majority of streams in our study area.

For streams exhibiting relatively constant flow the majority of the year, base flow can be estimated by visual examination of graphs of mean daily flow (S3). We constructed and examined graphs of mean daily flow and visually estimated base flow to provide additional confidence to our selected base flow values.

Due to the length of time that streams in our study areas spend in a base flow condition, and based on comparison to the available published base flow values, and examination of flow duration and mean daily flow graphs, we concluded that the 50^th^ percentile flow (median flow) is reflective of the base flow discharge.

**Delineating Study Watersheds**

We digitized the watershed area/ground water basin for each selected streamflow gage. ADWR basins or subbasins [4] were used as a starting point; borders were adjusted using watershed boundaries where appropriate (e.g. the Verde, Aqua Fria, upper Cienaga Creek, and Big Sandy watersheds). Selected gages correspond to the downstream point on study watersheds, with two exceptions: the Verde River near Clarkdale gage is used for the Verde River above Oak Creek study watershed and the Gila River head of Safford Valley gage is used for the Gila River at Calva study watershed. For these two study watersheds, an upstream gage was used because base flow in the lower reaches of the river was depleted due to irrigation diversions.

References:

1. Blasch KW, Hoffmann JP, Graser LF, Bryson JR, Flint AL (2006) Hydrogeology of the upper and middle Verde River watersheds, central Arizona: U.S. Geological Survey Scientific Investigations Report 2005–5198,102 p., 3 plates.
2. Santhi C, Allen PM, Muttiah RS, Arnold JG, Tuppad P (2008) Regional estimation of base flow for the conterminous United States by hydrologic landscape regions. Jour of Hydrology v 351 p 139-153.
3. Arizona Department of Water Resources (ADWR) )2009) Arizona Water Atlas, Draft Online Report. Available: <http://www.azwater.gov/AzDWR/StatewidePlanning/WaterAtlas/default.htm>. Accessed July 15, 2009.
4. Arizona Department of Water Resources (ADWR) )2009) Arizona Water Atlas, Draft Online Report. Available: <http://www.azwater.gov/AzDWR/StatewidePlanning/WaterAtlas/default.htm>. Accessed July 15, 2009.


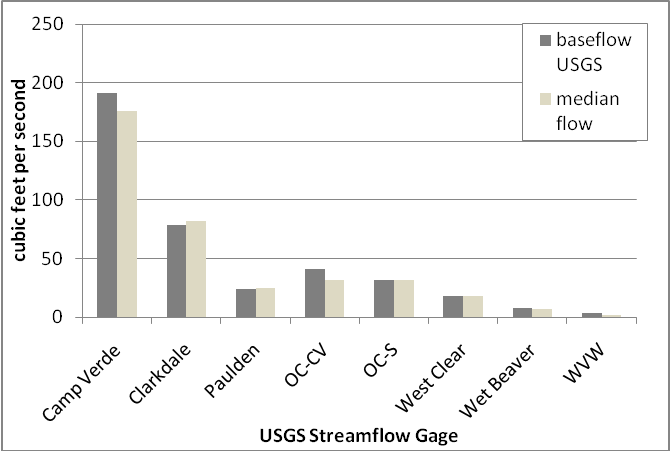


Figure S1. Comparison of published base flow values for USGS streamflow gages in the Verde watershed [3] and calculated median flow for those gages.


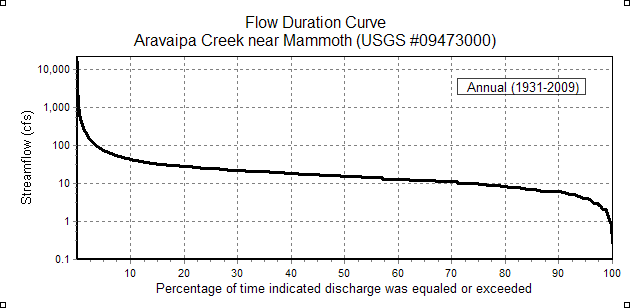
Figure S2A. Flow duration curve representative of small streams in study area.


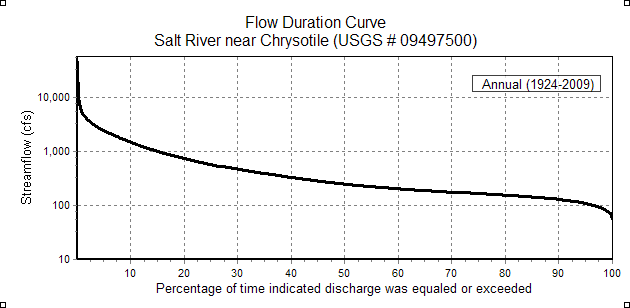


Figure S2B. Flow duration curve representative of large streams in study area.

Figure S3. Daily mean discharge, 2006, Verde River near Clarkdale (USGS # 09504000).
