# Supplementary material for: Sustainable Water Management in the Southwestern United States: Reality or Rhetoric?
Source: PLoS One. 2010 Jul 21;5(7):e11687. doi: 10.1371/journal.pone.0011687 (PMC2908145; doi:10.1371/journal.pone.0011687)
Supplement: Table S1 — Population, base flow, and municipal water demand under four scenarios for eighteen river basins in Arizona. Provides baseline data used for estimating population growth, water demand, river flows, and the values used in scenarios. (0.06 MB DOC) [file pone.0011687.s002.doc]

| Table S1. Population, Base Flow, and Municipal Water Demand Under Four Scenarios for Eighteen River Basins in Arizona. | | | | | | | | | |
| --- | --- | --- | --- | --- | --- | --- | --- | --- | --- |
| River Basin,  grouped by  base flow demand index^1^ | Census population 2000 | Water demand in gallons per-capita per-day | Base population projection 2050 | River base flow for historic period  (ac-ft/yr) | Projected 2050 municipal water demand in acre-feet/year under four population-water demand scenarios *(values in parenthesis indicate base flow demand index^1^)* | | | | |
|  |  |  |  |  | 2000 Baseline | Conservation & Low Growth | Conservation | Base Growth | High Growth |
| **BDI ≤ 50% of river base flow (under base growth projection)** | | | | | | | | | |
| Aravaipa Creek | 105 | 300 | 160 | 11,591 | *35 (0)* | 28 (*0)* | 38 (*0)* | 54 (*0)* | 67 (*1)* |
| Salt River | 11,077 | 128 | 23,995 | 111,565 | *1,588 (1)* | 1,806 (*2)* | 2,408 (*2)* | 3,440 (*3)* | 4,300 (*4)* |
| Burro Creek | 39 | 192 | 789 | 3,622 | *8 (0)* | 89 (*2)* | 119 (*3)* | 170 (*5)* | 212 (*6)* |
| Gila River | 39,366 | 135 | 56,064 | 127,503 | *5,953 (5)* | 4,451 (*3)* | 5,935 (*5)* | 8,478 (*7)* | 10,597 (*8)* |
| Oak Creek | 16,602 | 141 | 27,415 | 29,992 | *2,622 (9)* | 2,273 (*8)* | 3,031 (*10)* | 4,330 (*14)* | 5,412 (*18)* |
| Tonto Creek | 6,519 | 241 | 9,466 | 15,213 | *1,760 (12)* | 1,342 (*9)* | 1,789 (*12)* | 2,555 (*17)* | 3,194 (*21)* |
| Verde R. at Camp Verde | 124,753 | 141 | 290,675 | 138,804 | *19,704 (14)* | 24,102 (*17)* | 32,137 (*23)* | 45,909 (*33)* | 57,387 (*41)* |
| Williamson Valley Wash | 1,143 | 141 | 7,663 | 2,680 | *181 (7)* | 635 (*24)* | 847 (*32)* | 1,210 (*45)* | 1,513 (*56)* |
| **BDI 50.1-100% of river base flow (under base growth projection)** | | | | | | | | | |
| Upper Cienega Creek | 1,096 | 118 | 2,014 | 500 | *145 (29)* | 140 (*28)* | 186 (*37)* | 266 (*53)* | 33 (*67)* |
| Verde R. above Oak Ck. | 85,830 | 141 | 218,477 | 57,200 | *13,556 (24)* | 18,116 (*32)* | 24,154 (*42)* | 34,506 (*60)* | 43,133 (*75)* |
| Big Sandy River | 1323 | 192 | 10146 | 3,260 | *285 (9)* | 1146 (*35)* | 1527 (*47)* | 2,182 (*67)* | 2,728 (*84)* |
| **High > 100% of river base flow (under base growth projection)** | | | | | | | | | |
| Arivaca Creek | 848 | 182 | 1,672 | 304 | *173 (57)* | 179 (*59)* | 239 (*78)* | 341 (*112)* | 426 (*140)* |
| Little Colorado River | 84,076 | 147 | 154,592 | 22,458 | *13,844 (62)* | 13,364 (*60)* | 17,819 (*79)* | 25,455 (*113)* | 31,819 (*142)* |
| Upper Verde River | 60,304 | 135 | 166,905 | 17,677 | *9,119 (52)* | 13,251 (*75)* | 17,667 (*100)* | 25,239 (*143)* | 31,549 (*178)* |
| San Pedro River | 39,487 | 197 | 62,805 | 9,418 | 8,714 *(93)* | 7,276 (*77)* | 9,701 (*103)* | 13,859 (*147)* | 17,324 (*184)* |
| Lower Cienega Creek | 2,940 | 118 | 11,436 | 797 | *389 (49)* | 794 (*100)* | 1,058 (*133)* | 1,512 (*190)* | 1,889 (*237)* |
| Babocomari River | 17,602 | 197 | 25,209 | 536 | 3,884 *(725)* | 2,920 (*545)* | 3,894 (*726)* | 5,563 (*1,038)* | 6,954 (*1,297)* |
| Agua Fria River | 44,457 | 172 | 103,981 | 1,811 | 8,565 *(473)* | 10,518 (*581)* | 14,023 (*774)* | 20,033 (*1,106)* | 25,042 (*1,383)* |
| Base flow demand index (BDI) = water demand/base flow * 100. See materials and methods for further details.  2000 baseline = municipal water demand baseline in 2000; Conservationo & low growth = Population 25% less than base projection with demand reduced 30%; conservation = Base population projection with demand reduced 30%; base growth = Base population projection; high growth = Population 25% over base projection | | | | | | | | | |
|  | |  | | | | | | | |
