# Supplementary material for: Sustainable Water Management in the Southwestern United States: Reality or Rhetoric?
Source: PLoS One. 2010 Jul 21;5(7):e11687. doi: 10.1371/journal.pone.0011687 (PMC2908145; doi:10.1371/journal.pone.0011687)
Supplement: Table S2 — Vulnerable aquatic, riparian and wetland species found in 18 study watersheds in Arizona. Provides taxonomic and conservation data for the imperiled species found within our study watersheds. (0.09 MB DOC) [file pone.0011687.s003.doc]

| Table S2. Vulnerable aquatic, riparian and wetland species found in 18 study watersheds in Arizona. We selected species that occur within our study watersheds that could be classified as obligate or facultative wetland species [1] and are (1) federally designated under the US Endangered Species Act (endangered, threatened, candidate, proposed, special concern, similarity of appearance), or (2) designated as globally critically imperiled (< 5 viable populations), globally imperiled (6–20 viable populations), or globally vulnerable (21–80 populations), according to NatureServe global conservation status ranks. | | | | | |
| --- | --- | --- | --- | --- | --- |
| **Scientific Name** | | **Common Name** | **Global Conservation Status Rank** | **U.S. ESA Classification** | **% of U.S. Occur-rences in Study Area** |
| ***Amphibians & Reptiles*** | |  |  |  |  |
| Ambystoma tigrinum stebbinsi | | Sonoran Tiger Salamander | T1 | LE | 2 |
| Bufo microscaphus | | Arizona Toad | G3 |  | 42 |
| Hyla wrightorum pop. 2 | | Huachucas/Canelo Hills Treefrog | T2 | C | 33 |
| Rana chiricahuensis | | Chiricahua Leopard Frog | G3 | LT | 21 |
| Thamnophis rufipunctatus | | Narrow-headed Gartersnake | G3 |  | 59 |
| Birds | |  |  |  |  |
| Coccyzus americanus occidentalis | | Western Yellow-billed Cuckoo | T3 | C | 1 |
| Empidonax traillii extimus | | Southwestern Willow Flycatcher | T1 | LE | 10 |
| Haliaeetus leucocephalus pop. 3 | | Bald Eagle - Sonoran Desert Pop. | TNR | LT | 23 |
| Rallus longirostris yumanensis | | Yuma Clapper Rail | T3 | LE | 1 |
| ***Fish*** | |  |  |  |  |
| Agosia chrysogaster chrysogaster | | Gila Longfin Dace | T3 |  | 62 |
| Catostomus clarkii | | Desert Sucker | G3 |  | 37 |
| Catostomus insignis | | Sonora Sucker | G3 |  | 37 |
| Catostomus sp. 3 | | Little Colorado Sucker | G2 |  | 93 |
| Cyprinodon macularius | | Desert Pupfish | G1 | LE | 10 |
| Gila intermedia | | Gila Chub | G2 | LE | 79 |
| Gila nigra | | Headwater Chub | G2 | C | 55 |
| Gila robusta | | Roundtail Chub | G3 | C | 32 |
| Lepidomeda vittata | | Little Colorado Spinedace | G1 | LT | 96 |
| Meda fulgida | | Spikedace | G2 | LT | 34 |
| Oncorhynchus gilae | | Gila or Apache Trout | G3 | LT | 100 |
| Oncorhynchus gilae apache | | Apache Trout | T3 | LT | 25 |
| Poeciliopsis occidentalis occidentalis | | Gila Topminnow | T3 | LE | 24 |
| Ptychocheilus lucius | | Colorado Pikeminnow | G1 | LE, XN | 5 |
| Rhinichthys cobitis | | Loach Minnow | G2 | LT | 28 |
| Xyrauchen texanus | | Razorback Sucker | G1 | LE | 14 |
| ***Mammals*** | |  |  |  |  |
| Zapus hudsonius luteus | | New Mexican Jumping Mouse | T2 |  | 6 |
| ***Invertebrates*** | |  |  |  |  |
| Anodonta californiensis | | California Floater | G3 |  | 18 |
| Cicindela oregona maricopa | | Maricopa Tiger Beetle | T3 |  | 39 |
| Protoptila balmorhea | | Balmorhea Saddle-case Caddisfly | G2 |  | 100 |
| Psephenus montanus | | White Mountains Water Penny Beetle | G2 |  | 24 |
| Pyrgulopsis arizonae | | Bylas Springsnail | G1 |  | 100 |
| Pyrgulopsis glandulosa | | Verde Rim Springsnail | G1 |  | 100 |
| Pyrgulopsis montezumensis | | Montezuma Well Springsnail | G1 |  | 100 |
| Pyrgulopsis morrisoni | | Page Springsnail | G1 | C | 100 |
| Pyrgulopsis thompsoni | | Huachuca Springsnail | G2 | C | 64 |
| Sympetrum signiferum | | Spot-winged Meadowhawk | G2 |  | 20 |
| Tryonia gilae | | Gilae Tryonia | G1 |  | 100 |
| ***Vascular Plants*** | |  |  |  |  |
| Actaea arizonica | | Arizona Bugbane | G2 |  | 91 |
| Astragalus cobrensis var. maguirei | | Copper Mine Milk-vetch | T2 |  | 13 |
| Browallia eludens | | Elusive New Browallia Species | G2 |  | 100 |
| Carex chihuahuensis | | Chihuahuan Sedge | G3 |  | 29 |
| Carex ultra | | Cochise Sedge | G3 |  | 14 |
| Castilleja mogollonica | | White Mountains Paint Brush | G1 |  | 89 |
| Cirsium parryi ssp. mogollonicum | |  | T1 |  | 100 |
| Erigeron piscaticus | | Fish Creek Fleabane | G1 |  | 100 |
| Eryngium sparganophyllum | | Arizona Eryngo | G2 |  | 50 |
| Lilaeopsis schaffneriana var. recurva | | Cienega False Rush | T2 | LE | 40 |
| Lilium parryi | | Lemon Lily | G3 |  | 13 |
| Packera quaerens | | New Mexico Groundsel | G2 |  | 28 |
| Polemonium foliosissimum var. flavum | | Leafy Jacob's-ladder | T3 |  | 63 |
| Polemonium pauciflorum ssp. hinckleyi | | Hinckley's Jacob's-ladder | T2 |  | 46 |
| Rumex orthoneurus | | Blumer's Dock | G3 |  | 41 |
| Salix arizonica | | Arizona Willow | G2 |  | 33 |
| Salvia amissa | | Catalina Mountain Sage | G2 |  | 33 |
| Samolus vagans | | Chiricahua Mountain Brookweed | G2 |  | 25 |
| Spiranthes delitescens | | Canelo Hills Ladie's-tresses | G1 | LE | 75 |
| Symphyotrichum potosinum | | Santa Rita Mountain American-aster | G2 |  | 75 |
| Thelypteris puberula var. sonorensis | | Aravaipa Woodfern | T3 |  | 9 |
| Trifolium neurophyllum | | Mogollon Clover | G2 |  | 12 |
| Key to Abbreviations. | | | | | |
| Status under the U.S. Endangered Species Act | | | | | |
| LE | Listed as Endangered | | | | |
| LT | Listed as Threatened | | | | |
| C | Listed as Candidate Species | | | | |
| XN | Listed as non-essential experimental population | | | | |
| Status under the Heritage Program Global Species (G) and Sub-Species (T) Ranks | | | | | |
| G1 | Globally Critically Imperiled: five or less populations remaining globally | | | | |
| G2 | Globally Imperiled: six to 20 populations remaining globally | | | | |
| G3 | Globally Vulnerable: 21 – 80 populations remaining globally | | | | |
| T1 | Globally Critically Imperiled Sub-Species: five or less populations remaining globally | | | | |
| T2 | Globally Imperiled Sub-Species: six to 20 populations remaining globally | | | | |
| T3 | Globally Vulnerable Sub-Species: 21 – 80 populations remaining globally | | | | |

References:

1. U.S. Fish and Wildlife Service (USFWS). 1988 National list of vascular plant species that occur in wetlands: U.S. Fish & Wildlife Service Biological Report 88 (26.9).
